# Intra-aortic balloon pump placement in coronary artery bypass grafting patients by day of admission

**DOI:** 10.1186/s13019-020-01259-z

**Published:** 2020-08-14

**Authors:** Gabriel A. del Carmen, Andrea Axtell, David Chang, Serguei Melnitchouk, Thoralf M. Sundt, Amy G. Fiedler

**Affiliations:** 1 Department of Surgery, Massachusetts General Hospital, Harvard Medical School, Boston, MA USA; 2 Division of Cardiac Surgery, Massachusetts General Hospital, Harvard Medical School, Boston, MA USA; 3 Division of Cardiothoracic Surgery, University of Wisconsin, H4/320 CSC, 600 Highland Ave, Madison, WI 53792 USA

**Keywords:** Intra-aortic balloon pump, Coronary artery bypass grafting, Coronary artery disease

## Abstract

**Introduction:**

Intra-Aortic Balloon Pumps (IABPs) can be utilized to provide hemodynamic support in high risk patients awaiting coronary artery bypass grafting (CABG). There are many indications for IABP and institutional practice patterns regarding the placement of IABPs is variable. As a result, the preoperative placement of an IABP in a patient awaiting CABG is not standardized and may vary according to non-clinical factors. We hypothesize that the rate of IABP placement varies by day of the week.

**Methods:**

A retrospective cohort analysis of the Office of Statewide Health Planning and Development database from 2006 to 2010 was performed. All patients admitted for CABG were included. Patients who died within 24 h of admission and those who had absolute contraindications to IABP placement were excluded. The primary outcome was preoperative IABP placement versus non-placement. A multivariable logistic regression analysis to identify predictors of IABP placement was performed, adjusting for patient demographics, clinical factors, and system variables.

**Results:**

A total of 46,347 patients underwent CABG, of which 7695 (16.60%) had an IABP placed preoperatively. On unadjusted analysis, IABP rates were significantly higher on weekends versus weekdays (20.83% vs. 15.70%, *p* < 0.001). On adjusted analysis, patients awaiting CABG were 1.30 times more likely to have an IABP placed on weekends than on weekdays (OR: 1.30, 95% CI 1.20–1.40, *p* < 0.001).

**Conclusion:**

The odds of preoperative IABP placement prior to CABG is significantly increased on weekends compared to weekdays, even when controlling for clinical factors. Further exploration of this phenomenon and its associations are warranted.

## Introduction

It is estimated that 10–20% of all medical procedures performed may be medically unnecessary, subjecting patients to avoidable risks without any clinical benefit [1–3]. While clinical factors have been and continue to be studied, recent literature has identified various non-clinical factors that are increasingly recognized as playing a key role in the overall delivery of quality healthcare [4–7]. Treatment standardization, as it relates to both clinical and non-clinical factors, is a critically important metric in the quality of healthcare delivery so patients are not subjected to medically unnecessary risks [8].

Intra-Aortic Balloon Pumps (IABPs) are one of the most commonly used mechanical circulatory support devices to treat heart failure and cardiogenic shock [9]. IABPs can be inserted with ease in the cardiac catheterization laboratory via a percutaneous approach. The indications for placement of an IABP vary widely and are often institution-dependent. Notably, one indication for placement of an IABP is for prophylaxis in patients deemed high risk prior to undergoing surgical revascularization with coronary artery bypass grafting (CABG) [10]. The placement of an IABP in this situation falls to the clinical discretion of the interventional cardiologist and the cardiac surgeon. For example, an IABP may be placed in patients with concerning “anatomy” such as left main coronary artery stenosis or diffuse disease, despite the absence of clinical symptoms [11]. These patients will then remain in the intensive care unit with IABP hemodynamic augmentation until they undergo surgical revascularization. While IABP counter-pulsion provides hemodynamic support for patients with significant coronary artery disease, there are risks and complications associated with the placement and use of the device. These complications can range in severity from an ischemic lower extremity to the uncommon, yet devastating, complication of thrombosis of the descending thoracic aorta [12]. Because of the known clinical risks associated with the placement of IABPs, it is prudent to reduce medically unnecessary IABPs in order to minimize complications associated with the device, and shorten hospital length of stay.

While the public expects hospitals to be fully staffed regardless of the time of an operation, the reality is that staffing availability and societal pressures on medical staff may reduce a hospital’s ability to provide different complex operations during certain times of the week. Given the wide variability with respect to the timing and indication of IABP placement, specifically in the pre-operative patient with coronary artery disease (CAD) we believe the placement of IABPs in this patient population serves as an effective case study in the evaluation of a non-clinical factor that may influence clinical decision making. In this study, we chose to focus on influence of the day of the week on treatment decision. This has been noted in other fields in the literature; for example, Burns et al. found that the decision to deliver by Cesarean section differs based on the day of the week of the delivery, which is indicated by an increased rate of Cesarean deliveries on the weekend [13]. Given that IABPs may be placed for concerning coronary anatomy in a patient without symptoms yet requiring surgical revascularization, IABP placement is an ideal case scenario to determine how these rates change on weekends and weekdays. Therefore, we hypothesize that there will be an increased rate of preoperative IABP placement prior to CABG on Saturdays and Sundays when operating rooms have reduced capacity to perform non-scheduled cases.

## Materials and methods

A retrospective cohort analysis of the California Office of Statewide Health Planning and Development (OSHPD) was performed between 2006 and 2010. This database is maintained for all California-licensed facilities and captures all patients and payers and collects information on patients, treatments, and hospitals for every emergency department admission, inpatient admission/hospital discharge, outpatient visit, and ambulatory surgery. The study was approved by the Institutional Review Board at the Massachusetts General Hospital (16-05-2558).

Patients were identified and included using International Classification of Diseases, Ninth Revision, Clinical Modification (ICD9-CM) procedure codes [14]. We included all patients who underwent CABG in our analysis. Patients who died within 24 h of admission were excluded. In addition, all patients with an absolute contraindication to IABP placement, including occlusion or severe stenosis of the distal aorta, aortic aneurysm, aortic dissection, and severe aortic regurgitation, were excluded. The primary outcome was the preoperative placement of an IABP versus non-placement for patients awaiting CABG. Covariates considered included day of admission, age, race, teaching hospital status, type of hospital (i.e. rural, urban, or frontier), heart failure, coronary artery disease, acute coronary syndrome, and relative contraindications to IABP. These included severe peripheral vascular disease, aortic or iliofemoral bypass grafts, moderate aortic regurgitation, and sustained tachyarrhythmia. Unadjusted analysis was performed for categorical dependent variables with *χ* ^2^, and for continuous dependent variables with a Student’s t-test. A multivariable logistic regression model was constructed to identify independent risk factors for the odds of IABP placement. All covariates described above were included in the final model. Odds ratios (OR) are presented with 95% confidence intervals (CI). All analyses were completed using STATA v13.1 (STATA Corp., Texas, USA). A *p*-value of less than 0.05 was considered statistically significant.

## Results

A total of 46,347 patients underwent CABG, of which 8148 (18%) were admitted on the weekend (Saturday/Sunday) and 38,199 (82%) on a weekday (Monday-Friday). Baseline characteristics of the study population are presented in Table 1. Among all patients undergoing CABG approximately 60% were white, 4% were black, 18% were Hispanic, and 10% were Asian. Over a third (41%) were greater than 70 years of age and the majority were treated in non-teaching hospitals (73%). A total of 7695 (16.60%) had an IABP placed pre-operatively.

**Table 1:** Baseline characteristics of all patients undergoing CABG

Patient Characteristics (***n*** = 46,347)	n	(%)
**Age Category**		
70+ age	17,440	40.72
60–69 age	12,817	29.93
50–59 age	9213	21.51
40–49	2945	6.88
18–39	413	0.96
**Race**		
White	27,981	61.95
Black	1870	4.14
Hispanic	8264	18.30
Asian	4797	10.62
Other	2254	4.99
**BMI Class**		
Not Coded	37,095	80.04
BMI 25–29.9	6700	14.46
BMI > 30	2552	5.51
**Insurance**		
Private Coverage	14,237	30.72
MediCal	4855	10.48
Medicare	23,227	50.12
Self-Pay	1366	2.95
Other Non-Federal Indigent	1557	3.36
Other	1103	2.38
**Day of Admission**		
Weekend	8148	17.58
Weekday	38,199	82.42
**Heart Failure**	15,204	32.80
**Severe Peripheral Vascular Disease**	6094	6.99
**Aortic/Iliofemoral Bypass Grafts**	590	0.68
**Aortic Regurgitation**	88	0.10
**Sustained Tachyarrhythmias**	4685	5.38
**IABP**	7695	16.60
**Hospital Characteristics**		
**Beds**		
228+ Beds	38,759	84.71
122–227 Beds	6060	13.24
52–121 Beds	553	1.21
< 52 Beds	384	0.84
**Urbanicity**		
Urban	1268	2.74
Non-Urban	45,079	97.26
**Teaching Status**		
Teaching	12,431	26.82
Non-Teaching	33,916	73.18
**Hospital Volume**		
< 199 Patients	25,797	55.84
200–399 Patients	12,478	27.01
400–699 Patients	4536	9.82
> 700 Patients	3383	7.32

*BMI* Body Mass Index; *CABG* Coronary Artery Bypass Grafting; *IABP* Intra-Aortic Balloon Pump

On unadjusted analysis (Table 2), 15.70% of all CABG patients who had an IABP placed were admitted on a weekday and 20.83% were admitted during the weekend (*p* < 0.001). There was no difference in age, race, BMI, and hospital characteristic (teaching v. private) between patients who had an IABP placed on a weekend compared to those who had an IABP placed on a weekday (all *p* < 0.001). Patients covered under Medicare made up a greater percentage of the total patient populations on the weekends relative to the weekdays (49.56% vs. 32.59%, *p* < 0.001).

**Table 2:** Baseline characteristics of CABG patients by day of admission

Patient Characteristics (***n*** = 46,347)	n	(%)
**Age Category**		
70+ age	17,440	40.72
60–69 age	12,817	29.93
50–59 age	9213	21.51
40–49	2945	6.88
18–39	413	0.96
**Race**		
White	27,981	61.95
Black	1870	4.14
Hispanic	8264	18.30
Asian	4797	10.62
Other	2254	4.99
**BMI Class**		
Not Coded	37,095	80.04
BMI 25–29.9	6700	14.46
BMI > 30	2552	5.51
**Insurance**		
Private Coverage	14,237	30.72
MediCal	4855	10.48
Medicare	23,227	50.12
Self-Pay	1366	2.95
Other Non-Federal Indigent	1557	3.36
Other	1103	2.38
**Day of Admission**		
Weekend	8148	17.58
Weekday	38,199	82.42
**Heart Failure**	15,204	32.80
**Severe Peripheral Vascular Disease**	6094	6.99
**Aortic/Iliofemoral Bypass Grafts**	590	0.68
**Aortic Regurgitation**	88	0.10
**Sustained Tachyarrhythmias**	4685	5.38
**Acute Coronary Syndrome**	12,833	27.69
**IABP**	7695	16.60
**Hospital Characteristics**		
**Beds**		
228+ Beds	38,759	84.71
122–227 Beds	6060	13.24
52–121 Beds	553	1.21
< 52 Beds	384	0.84
**Urbanicity**		
Urban	1268	2.74
Non-Urban	45,079	97.26
**Teaching Status**		
Teaching	12,431	26.82
Non-Teaching	33,916	73.18
**Hospital Volume**		
< 199 Patients	25,797	55.84
200–399 Patients	12,478	27.01
400–699 Patients	4536	9.82
> 700 Patients	3383	7.32

*BMI* Body Mass Index; *CABG* Coronary Artery Bypass Grafting; *IABP* Intra-Aortic Balloon Pump

On multivariable logistic regression modeling (Table 3), the odds of having an IABP placed on a weekend increased relative to having one placed on a weekday (OR: 1.30, CI: 1.21–1.40, *p* < 0.001). Patients with acute coronary syndrome were less likely to receive an IABP than patients without the morbidity (OR: 0.48, CI: 0.44–0.53, *p* < 0.001). Although acute coronary syndrome is an indication for IABP placement, the non-inclusion of non-emergent cases is likely responsible for this observed effect. Black patients were less likely to have an IABP than whites (OR 0.91, CI: 0.68–0.95 *p*-value 0.012). Interestingly, we found that the likelihood of preoperative IABP placement was significantly less likely in non-teaching hospitals than teaching hospitals (OR: 0.67, CI: 0.48–0.93, p 0.018). When considering the likelihood of in-hospital mortality, patients who had an IAPB placed preoperatively had 5 times higher odds of mortality than those who did even when controlling for all the covariates in our logistic model (OR: 5.00, CI: 4.30–5.82, *p* < 0.001). This may be related to the variety of clinical indications for which an IABP is placed, as well as physician judgment which cannot be controlled for in a retrospective study. Sicker patients, for example, while more likely to receive IABPs, are also more likely to fare worse outcomes. Patients treated at non-teaching hospitals had an increased odds of in-hospital mortality compared to those treated at teaching hospitals (OR: 1.33, CI: 1.13–1.56, *p* = 0.001). MediCal and Medicare patients were also more likely to have an in-hospital mortality compared to those with private insurance (OR: 1.66, CI: 1.29–2.14, *p* < 0.001 and OR: 1.50, CI: 1.24–1.82, *p* < 0.001 respectively). To account for the increased proportion of patients admitted for heart failure over the weekend, we performed a subset analysis without the heart failure patient population. We found that the proportion of heart failure patients made no qualitative difference on the likelihood of receiving an IABP on the weekend relative to the weekday (OR: 1.30, CI: 1.22–1.38, *p* < 0.001).

**Table 3:** Multivariable logistic regression for odds of IABP placement

Factor	Odds Ratio	95% Confidence Interval	***p***-value
**Day of Admission**			
Weekday	Reference		
Weekend	1.30	1.21–1.40	< 0.001
**Age Category**			
> 70 age	Reference		
60–69 age	1.06	0.97–1.15	0.221
50–59 age	1.06	0.95–1.17	0.320
40–49 age	1.09	.94–1.27	0.245
18–39 age	1.79	1.41–2.28	< 0.001
**Race**			
White	Reference		
Black	0.91	0.68–0.95	0.012
Hispanic	1.04	0.93–1.16	0.510
Asian	1.10	0.98–1.23	0.095
Other	1.03	0.73–1.45	0.875
**Insurance**			
Private Coverage	Reference		
MediCal	1.01	0.84–1.22	0.900
Medicare	0.89	0.77–1.04	0.163
Self-Pay	1.28	1.02–1.61	0.031
Other Non-Federal Indigent	0.95	0.75–1.22	0.706
Other	1.21	0.81–1.82	0.352
**BMI Class**			
Not Coded	Reference		
BMI 25–29.9	0.89	0.80–0.98	0.022
BMI > 30	0.90	0.79–1.01	0.081
**Heart Failure**	2.17	2.09–2.48	< 0.001
**Severe Peripheral Vascular Disease**	0.66	0.59–0.75	< 0.001
**Aortic/Iliofemoral Bypass Grafts**	1.6	1.10–2.34	0.015
**Aortic Regurgitation**	0.73	0.26–2.04	0.549
**Sustained Tachyarrhythmias**	1.96	1.77–2.17	< 0.001
**Acute Coronary Syndrome**	0.48	0.44–0.53	< 0.001
**Hospital Characteristics**			
**Beds**			
228+ Beds	Reference		
122–227 Beds	1.23	0.66–2.31	0.509
52–121 Beds	1.15	0.82–1.62	0.43
< 52 Beds	0.94	0.82–1.08	0.404
**Urbanicity**			
Urban	Reference		
Non-Urban	1.27	0.91–1.79	0.164
**Teaching Status**			
Teaching	Reference		
Non-Teaching	0.67	0.48–0.93	0.018
**Hospital Volume**			
< 199 Patients	Reference		
200–399 Patients	0.79	0.63–1.01	0.055
400–699 Patients	0.43	0.33–0.56	< 0.001
> 700 Patients	0.49	0.41–0.58	< 0.001

*BMI* Body Mass Index; *IABP* Intra-Aortic Balloon Pump

## Discussion

In this study, we found that the rate of preoperative IABP placement in patients awaiting CABG was significantly higher when the patient was admitted on a weekend (Saturday/Sunday) compared to a weekday. To our knowledge, this is the first study to investigate the association between day of admission and the rate of IABP placement.

Variation as an indicator of healthcare quality is a novel goal of investigation, with the aim of improving patient-centered care by removing variability based on non-clinical factors. There have been few examples looking at variations in practice patterns by non-clinical factors. The most notable example is from the *Dartmouth Atlas of Healthcare* which found significant variations in practice patterns across geographic regions in the U.S. [15] Similarly, variations in Cesarean section delivery rates were found across different days of the week [13]. Many factors have been speculated to cause practice pattern variations along non-clinical factors, such as convenience, financial incentive, market competitions, and so on. Most of this literature focuses on procedures that are discretionary and less invasive. We extend this line of investigation to cardiac surgery which, as a complex procedure, would be thought to be controlled under strict clinical guidelines and not be influenced by non-clinical factors.

Previous studies have investigated the effect of day of admission on the clinical outcomes of various procedures. However, the literature is mixed regarding surgical outcomes when comparing weekend to weekday admission. For example, Baid-Agrawal and colleagues examined the outcomes of renal transplantation when performed on a weekend versus a weekday using the UNOS database. They concluded that the outcomes for deceased donor kidney transplantation in the US were not affected by the day of surgery [16]. This is in contrast to the findings of Glance and colleagues who utilized the Healthcare Cost and Utilization Project Nationwide Inpatient Sample (HCUP NIS) to evaluate patients undergoing major surgeries, including CABG, to determine if they were more likely to die or experience a major complication when the surgery was performed on a weekend compared to a weekday. The investigators determined that patients undergoing non-emergent major cardiac and non-cardiac surgery on a weekend had a significantly increased risk of death and major complications compared to those undergoing surgery on a weekday [15]. This raises the question of potential system-based and non-clinical factors associated with a “weekend effect.” Our study differs and expands on this work by focusing on variations in the rate of procedure, in addition to clinical complications and mortality. Variation itself is the primary outcome because it indicates a lack of standardization in clinical practice for reasons not explained by a specific clinical indicator.

Our focus on non-clinical variation as an outcome is critical in improving the quality of care that patients receive. Placement of IABPs are associated with many risks, such as major limb ischemia and mortality [17]. Medically unnecessary procedures subject patients to risks with no clinical benefit. Ensuring the appropriateness of any clinical procedure is vital to improving healthcare quality. The existence of this variation in IABP procedure rates among days of the week indicates that the application of an invasive procedure is not only widely variable amongst institutions, but is highly subject to non-clinical factors and is impacted by day of week variability. Healthcare decisions should be evidence-based and patient centered. It is important that non-clinical factors are minimized in the administration of healthcare.

The need to minimize non-clinical factors is highlighted by several interesting ancillary findings found in this study. For example, we found evidence of disparity along race and insurance status. Black populations were less likely to receive IABPs compared to white populations despite no difference in clinical presentation. We also found that insurance status was a predictor for IABP placement. This is consistent with a growing body of literature on surgical disparities. Even though the investigation into these other disparities is beyond the scope of this study, the existence of these non-clinical influences underlines a concern regarding the influence of non-clinical factors on practice patterns.

This study has certain strengths and limitations. One major strength is the large sample size captured by use of a statewide database. The OSHPD database is powerful and allowed us to evaluate a wide range of both clinical and non-clinical data over a five-year period. This afforded us the ability to exclude comorbidities that serve as clear clinical contraindications to placement of IABP. Because of the large and diverse population, we could control for a variety of different factors which were further stratified to determine significant differences between groups. This study is subject to the inherent limitations of a retrospective database analysis. Large administrative databases often lack clinical granularity and there is a potential for a substantial amount of residual confounding. This precludes us from making sweeping conclusions about the nature of our findings and makes salient the need for further investigations. These investigations must capture other relevant and individual information that was not captured in OSHPD, such as STS scores, urgency of clinical intervention, and any other relevant and individualized clinical influences that cannot be captured on a broader scale. Similarly, because OSHPD does not provide the data necessary to distinguish between emergent, elective, and urgent patient cases, this study was unable to stratify on the basis of severity and urgency of cases. Additionally, as this topic is subject to the bias of surgeon and interventional cardiologist, a specific limitation is the inability to account for physician preference and practice technique which may confound the results. This is an important consideration, as there may be a variety of factors which may preclude a patient from undergoing an operation on a weekend, including surgeon preference, operating room time and staff availability, and hospital policies. Furthermore, the age of the data (2006–2010) available from the database limits the scope of our findings and warrants further investigations to determine if this trend has held. These types of factors will be critical to understand in future investigations to quantify and identify practice pattern variability based on weekday versus weekend admission. In addition, it will be important to investigate if financial or patient outcomes are affected by these decisions. We were also unable to account for a potential “weekday bias” among patients. We were unable to measure whether patients with less-serious cases do not admit themselves into the hospital on weekends, which would therefore create a weekend population of patients who present a more intense morbidity and therefore cannot avoid going to the hospital. Therefore, further qualitative research should investigate the role of patient behaviors on this weekend effect to determine the best way to standardize the procedure.

Our study has important implications. The discovery of this trend indicates the need for further investigations into the clinical reasons given for placement of IABP and may reflect an underlying disagreement with current practice guidelines. Future guideline refinement should ensure broad-based input in the development process to ensure larger buy-in, and thus broader compliance. It has been shown that the process by which consensuses are developed may influence the results and acceptability of the results. For example, details such as how consensus is defined, how disagreement is handled, and how sensitive the group is to process issues may all impact the ultimate acceptability of the proposed guideline. Additionally, given our findings, it is important to suggest guidelines for placement of IABP. Patients should have IABP placed if they present with unstable angina, active and on-going chest pain, cardiogenic shock, and have favorable femoral arterial anatomy for placement of IABP. IABP should not be placed due to concern over high risk anatomy, in unfavorable femoral arterial configurations, or in patients without active chest pain on presentation. Inasmuch, the care of patients who present with cardiac conditions has increasingly become more team based and multidisciplinary. It is important to assess patients on an individualized basis, and in those with coronary disease, utilize the institutional heart team to ensure the highest quality care. Using this multi modality approach ensures that the patient not only receives the most appropriate pre-procedural care, but also the best revascularization strategy, be it surgical or PCI for the patient.

## Conclusion

In conclusion, we demonstrate that there is a statistically significant increase in the placement of preoperative IABPs for patients awaiting CABG on the weekends compared to the weekdays. Further study elucidating the exact reasons for this variability are warranted. Creating and adhering to specific clinical guidelines regarding IABP placement may also reduce this unwanted variation.

## Data Availability

Available upon request.

## Abbreviations

IABP: Intra-aortic balloon pump
CAD: Coronary artery disease
CABG: Coronary artery bypass grafting
